# Impact of selective digestive decontamination on the pangenome composition of ESBL-*E. coli*

**DOI:** 10.1093/jac/dkag223

**Published:** 2026-06-23

**Authors:** Julian A Paganini, Anita C Schürch, Jelle Scharringa, Marc J M Bonten, Rob J L Willems, Nienke L Plantinga

**Affiliations:** Department of Medical Microbiology, University Medical Center Utrecht, Utrecht, The Netherlands; Department of Medical Microbiology, University Medical Center Utrecht, Utrecht, The Netherlands; Department of Medical Microbiology, University Medical Center Utrecht, Utrecht, The Netherlands; Julius Center for Health Sciences and Primary Care, University Medical Center Utrecht, Utrecht University, Utrecht, The Netherlands; Department of Medical Microbiology, University Medical Center Utrecht, Utrecht, The Netherlands; Department of Medical Microbiology, University Medical Center Utrecht, Utrecht, The Netherlands

## Abstract

**Objectives:**

Selective digestive decontamination (SDD) is routinely applied in Dutch ICUs to prevent colonization by potentially pathogenic microorganisms. In the R-GNOSIS ICU study, conducted outside of the Netherlands, SDD consisted of a mix of an oropharyngeal paste and a gastric suspension containing colistin, tobramycin and nystatin. Although SDD improves patient outcomes, its impact on the pangenome and resistome of colonizing *Escherichia coli* remains poorly understood. This study aimed to determine whether SDD influences the genomic composition and resistance repertoire of *E. coli* isolates from ICU patients.

**Methods:**

We compared 129 genomes of *E. coli* isolates from patients that received SDD and patients that did not receive SDD, but standard care only (baseline patients) in five ICUs located across three European countries (R-GNOSIS ICU study). Comparative analyses were performed to assess differences in the pangenome, plasmidome and antibiotic resistance gene content between groups.

**Results:**

The overall pangenome compositions of *E. coli* isolates from SDD-treated and baseline patients were highly similar. Accessory genome variation was strongly associated with phylogeny, but not with SDD exposure. Plasmidome differences were explained by the interaction of ICU location and phylogroup. A tobramycin resistance gene, flanked by IS26 elements and frequently co-occurring with *bla*_CTX-M-15_, was more prevalent in isolates from SDD patients. No *mcr* genes associated with transferable colistin resistance were detected.

**Conclusions:**

SDD did not significantly alter the overall pangenome or plasmidome composition of colonizing *E. coli* in ICU patients. However, a potentially mobile tobramycin resistance gene was more prevalent in *E. coli* from SDD patients.

## Introduction

In the Netherlands, patients admitted to the ICU and undergoing mechanical ventilation receive selective digestive decontamination (SDD) as a prophylactic treatment to prevent colonization with potentially pathogenic microorganisms (PPMOs). SDD includes topical tobramycin, colistin, amphotericin B (paste + nasogastric solution), plus a 4 day course of intravenous cephalosporin.^1–3^ A variation of SDD, named selective oropharyngeal decontamination (SOD), consisting only of the oropharyngeal paste, is administered in some ICUs as an alternative to SDD.^4^ In the Netherlands, resistance prevalence is low,^5^ SDD improved patient outcomes compared to standard care, reducing mortality, ICU stay and ICU-acquired bacteremia.^6–9^

The R-GNOSIS ICU study, conducted between 2013 and 2017, compared the effectiveness of SDD, SOD, chlorhexidine 1% mouthwash and standard care (baseline) in 13 ICUs located in six European countries with medium to high prevalence of antibiotic resistance (defined as having an extended-spectrum β-lactamase—ESBL—prevalence of at least 5% of amongst ICU-acquired bacteremia with Enterobacteriaceae).^10^ In this cluster-randomized trial, each treatment was applied during 6 months in the entire ward (randomized order), to patients with expected length of mechanical ventilation of at least 24 h. An important modification of the SDD regime in this study was the absence of the 4 day course of intravenous cephalosporin.

One concern is that SDD adds antibiotic pressure in ICUs, which already have the highest levels of antimicrobial use within hospitals.^11^ Yet, multiple studies suggest that SDD is associated with a decrease in incidence of colonization and infection with resistant microorganisms.^12–14^ On the other hand, a study based on metagenomic data seems to indicate that resistance genes to three classes of antibiotics, namely aminoglycosides, macrolides and tetracyclines, are more abundant in the gastrointestinal tract of SDD-treated patients when compared to healthy individuals.^15,16^ In addition to the potential selective effects on resistance genes, studies also indicate that SDD treatment alters the gut microbiome composition of ICU patients.^17,18^ These ecological changes of the microbiota may also affect the composition of PPMOs, such as *Escherichia coli*, populating the intestinal tract of patients receiving SDD. However, phenotypic profiling and conventional short-read metagenomics largely provide community- or gene-level summaries and often cannot assign antimicrobial resistance genes (ARGs) to the bacterial lineages and genetic contexts that harbour them, or separate clonal replacement from horizontal dissemination.^19–21^ Therefore, we hypothesize that SDD treatment shapes the pangenome composition of *E. coli*, including the development of resistance.

To address this question, we have sequenced the genomes of 129 *E. coli* isolates from the R-GNOSIS ICU study. These isolates were obtained from patients that received either SDD (*n* = 63) or did not receive SDD (*n* = 66, baseline) in five different ICUs located in Spain, Belgium and the UK. We explored the population structure of these isolates and compared their accessory genome content, plasmidomes and resistomes to determine if SDD leads to the selection of specific genomic features of *E. coli*.

## Materials and methods

### R-GNOSIS ICU study and selection of isolates for whole genome sequencing

The R-GNOSIS ICU study was conducted between December 2013 and May 2017 in 13 ICUs from six European countries. A detailed description of the study’s aims and methods can be found in.^10^ Briefly, to monitor the effect of SDD on colonization with Gram-negative bacteria, surveillance samples were taken twice weekly from the rectum and respiratory tract of all patients included in the study (*n* = 8496). Samples were inoculated on ESBL selective media (Biomerieux®) and in case of growth, phenotypic susceptibility testing was performed according to local standard operating procedures (for colistin susceptibility testing, *E*-tests were provided).^10^ Clinical blood and respiratory samples were obtained at the discretion of the clinician and processed according to local laboratory protocols. From these cultures, unique highly resistant microorganisms were stored for whole genome sequencing (WGS) according to the following rule: per patient, per body site, each first *E. coli*, with a unique phenotypic resistance pattern.

We submitted for WGS all stored *E. coli* isolates from the SDD and baseline periods of the five hospitals with the most stored isolates (AN, PS, UZ, LB and CD). Baseline isolates were defined as those collected from patients who did not receive SDD treatment during the study. Isolates were only included if they were collected from Day 2 of inclusion onwards (with the date of study enrolment being Day 0), to ensure sufficient exposure to the antimicrobials used in SDD. Metadata associated with sequenced isolates can be found in Data S1 (available as Supplementary data at *JAC* Online).

### Whole genome sequencing

Selected isolates were sequenced using Illumina MiSeq, with a Nextera XT pair-end kit (2 × 150 bp). Short reads were quality-trimmed with trim-galore (v0.6.6) (https://github.com/FelixKrueger/TrimGalore). Genomes were assembled using Unicycler^22^ (v0.4.9) and assembly quality was evaluated using QUAST^23^ and BUSCO.^24^ Across the 129 isolates, sequencing and assembly quality were consistently high: the median number of raw reads per isolate was 1 542 795 (IQR, 1 235 138–2 047 127), of which 99.93% were retained after trimming (IQR, 99.92%–99.94%). Assemblies had a median size of 5.04 Mb (IQR, 4.91–5.17 Mb), median N50 of 152 881 bp (IQR, 113 192–197 361 bp), median L50 of 11 (IQR, 9–14) and median BUSCO single-copy genes of 99.4% (IQR, 99.3%–99.4%). Raw reads were deposited in the SRA database. SRA accessions, trimming statistics and assembly quality metrics for all isolates are provided in Data S1.

### Pangenome analysis

Genomes were first annotated with BAKTA (v1.6.1).^25^ Panaroo^26^ was then used to define core and accessory genes. A core gene was defined as being present in 99% of all sequenced isolates.

Using the presence/absence gene matrix generated by Panaroo, we calculated Jaccard distances between accessory genomes of all pairs of isolates as:


$$JaccardDistance=1-\frac{Acc.genesofIsolate1\cap Acc.genesofIsolate2}{Acc.genesofIsolate1\cup Acc.genesofIsolate2}$$

Pangenome accumulation curves were obtained using the R micropan package.^27^

### Population structure determination

Multi-locus sequence type of isolates was predicted *in silico* with mlst (v2.1.6) (https://github.com/tseemann/mlst). Phylogroups were predicted with ClermonTyping^28^ (v20.03) (https://github.com/A-BN/ClermonTyping). PopPUNK (v2.4)^29^ was used to assign draft genomes to existing clusters according to the *E. coli* database (v1) available at https://www.bacpop.org/poppunk-databases.

A neighbour-joining tree was constructed using IQ-TREE^30^ (v2.2.0.3), based on a core-genome alignment obtained with Panaroo.

### Plasmidome analysis

Detailed procedures for plasmidome analysis, plasmid reconstructions and plasmid copy number estimation are provided in the Supplementary material.

### Comparison of plasmidome and accessory genomes

Distances between accessory genomes and plasmidomes of isolates were visualized using the t-distributed stochastic neighbour embedding algorithm (t-SNE), as implemented in the Rtsne R package (v0.15).

To conduct permutational analysis of variance (PERMANOVA), we used the adonis function from the vegan R package (v2.5-6) using the matrix of pairwise Jaccard distances as input. To explain the variance of accessory genome and plasmidome distances, six different PERMANOVA models were built with different explanatory variables each, as detailed in Tables S1 and S2. In models with two variables, interaction terms between them are indicated with ‘*’.

### ARGs, co-occurrence networks and genomic context

Antibiotic resistance genes were identified by using AMRFinderPus^31^ (v3.11.2). Co-occurence of ARGs in the same plasmids was calculated by using a previously described approach.^32^ Genomic context reconstruction of Tn(TobraR)/*bla*_CTX-M-15_ and read-based screening for the *rrsB* T1406A mutation were performed as described in the Supplementary material.

### Statistics and code availability

Comparison of medians was performed using the non-parametric test Wilcoxon rank-sum test^33^ and Fisher’s exact test,^34^ respectively. All statistical analysis was performed using R (v3.6.1), and code needed to reproduce this analysis can be found in https://gitlab.com/jpaganini/rgnosis_sdd_baseline.

## Results

### Patients colonized with ESBL-*E. coli* in five European ICUs

The five selected ICUs (AN, PS, UZ, LB and CD) were located in five different hospitals in Belgium (*n* = 3), Spain (*n* = 1) and the UK (*n* = 1). In total, 129 isolates were obtained from 116 patients, and in most cases (*n* = 103), a single isolate per patient was sequenced. Most sequenced isolates derived from the ICU were termed LB (*n* = 55), while the rest of the isolates were similarly distributed across the remaining locations, ranging from 16 (PS) to 21 (AN) isolates per ICU (Table 1). There was a similar number of isolates from SDD (*n* = 63) and baseline (*n* = 66) periods. The majority of isolates (*n* = 124, 96.1%) were obtained from rectal swabs, while a small number derived from respiratory samples (*n* = 4, 3.1%) and bloodstream infections (*n* = 1, 0.8%). Also, the majority of isolates (*n* = 122, 94.6%) were phenotypically resistant to cefotaxime, ceftriaxone and/or ceftazidime. We refer to the isolates as ESBL-*E. coli, a*lthough not all isolates have phenotypic ESBL confirmation. The median time between the start of SDD and the culture date of the sequenced ESBL-*E. coli* was 4 days (IQR = 2–6.5), while for baseline samples, the median time was 6 days (IQR = 4–11) after inclusion. All metadata associated with patients and sequenced isolates can be found in Data S1.

**Table 1.** Sequenced isolates according to study period, hospital and time of isolation

			Baseline	SDD	Total
Nr. of sequenced isolates	Per hospital	LB	28	27	55
		CD	13	7	20
		PS	5	11	16
		AN	14	7	21
		UZ	6	11	17
	Per body site	blood	1	0	1
		rectum	62	62	124
		respiratory	3	1	4
Median nr. of days until sample (IQR)			6 (4–11)	4 (2–6.5)	

### Population structure of colonizing ESBL-*E. coli*

**Figure 1.**
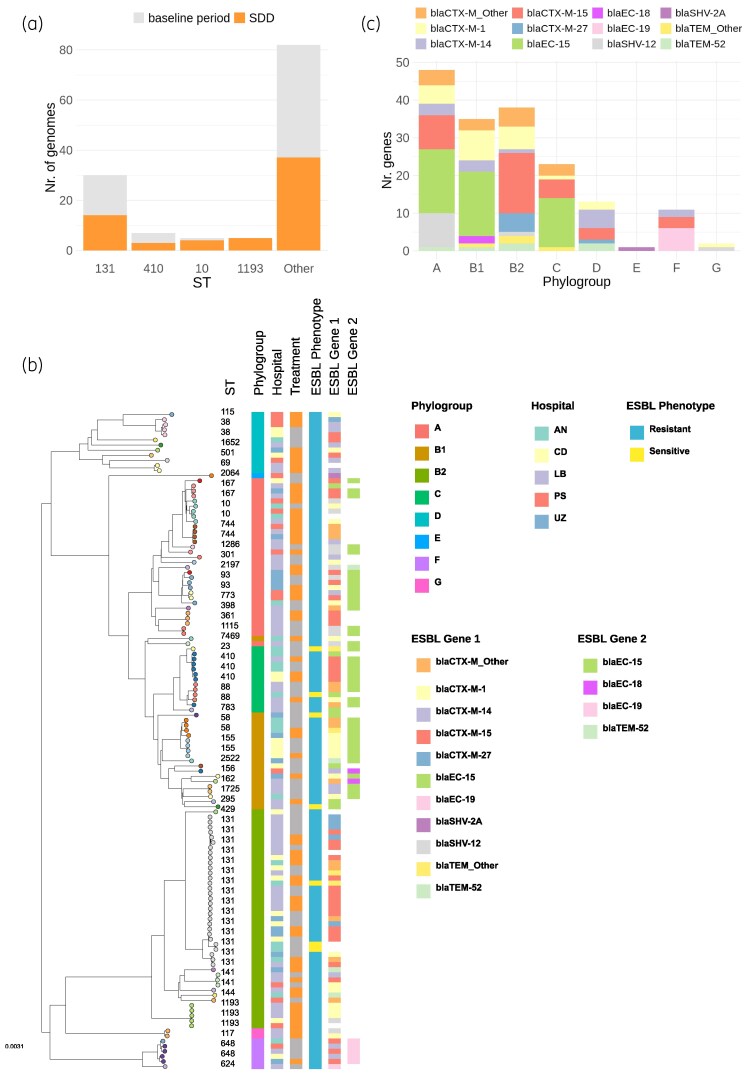
(a) Distribution of most abundant ST across study periods. Groups with less than five isolates were collapsed into the ‘Other’ category. (b) Neighbour-joining phylogenetic tree constructed based on core-genome alignment. Labels on the leaves indicate ST of isolates. Phylogroups were predicted in silico using ClermonTyper. ESBL Genes were predicted with AMRFinderPlus, if a second ESBL gene was present in an isolate, this is indicated in the column ESBL_Gene_2. ESBL phenotype indicates phenotyipic resistance or sensitivity to third-generation cephalosporins, evaluated as indicated in Materials and methods. (c) Distribution of the different ESBL genes across phylogroups.

Sequenced isolates belonged to 54 different STs, 11 of these were present in both study periods, 24 STs were only found in isolates from the baseline period and 19 were exclusively found in SDD patients. ST131 was the predominant clone (*n* = 30) in both groups (baseline *n* = 16, 24.2%; SDD *n* = 14, 22.2%) (Figure 1a), followed by ST410 (*n* = 7), ST10 (*n* = 6) and ST1193 (*n* = 5). We used PopPUNK to assign isolates to existing clusters considering both core and accessory genome variations (Data S1). We found a total of 53 clusters, of which the most abundant was Cluster 2 (*n* = 27, 21%), which was entirely composed of ST131 isolates. Cluster 7_510, the second most abundant (*n* = 12, 9.3%), encompasses isolates from ST10 (*n* = 4), ST167 (*n* = 3), ST744 (*n* = 4) and ST1695 (*n* = 1). A more detailed exploration of the core-genome of isolates (Figure 1b) showed no clear clusters associated with treatment, suggesting that SDD does not select for particular *E. coli* clones or lineages.

Simpson’s indices of diversity calculated using ST (baseline = 0.919, SDD = 0.921) and PopPUNK clusters (baseline = 0.924, SDD = 0.918) showed that the population structure in both study periods was equally diverse.

Predictions of ARGs from our dataset showed that most isolates (*n* = 123, 95.3%) carried at least one ESBL gene (Figure 1b). All isolates classified as phylogroup B2 (*n* = 42) carried only one ESBL gene, with *bla*_CTX-M-15_ being the most abundant allele (*n* = 16, 38.1%), followed by *bla*_CTX-M-1_ (*n* = 6, 14.3%) and *bla*_CTX-M-27_ (*n* = 5, 11.9%) (Figure 1b and c). Similarly, samples belonging to phylogroup D (*n* = 12) also carried one ESBL gene, but *bla*_CTX-M-14_ was the most prevalent variant (*n* = 5, 41.6%). In contrast, isolates from phylogroups A (*n* = 17/30), B1 (*n* = 15/20) and C (*n* = 10/13) carried two ESBL genes, and *bla*_EC-15_ was the most frequent gene in all phylogroup, with prevalences of 35%, 48% and 57%, respectively.

### Variations in accessory genome compositions are associated with phylogroup and hospital, but not with the use of SDD

Considering all 129 isolates, the total pangenome consisted of 13 753 genes, of which 3072 were identified as core- and the remaining 10 681 as accessory genes. Accumulation curves fitted to Heap’s law for baseline and SDD isolates yielded similar alpha values (baseline = 0.87, SDD = 0.91) (Figure S1A), confirming an open pangenome for both groups.^35^ The number of accessory genes in isolates from the baseline (median = 1,645, IQR = 1503–1776) and SDD periods (median = 1,642, IQR = 1476–1775) did not differ (*P* value = 0.92, Wilcoxon rank-sum test) (Figure S1B).

To identify associations between gene frequency and function, we obtained the functional categories of Clusters of Orthologous Groups (COG) from BAKTA annotations. A COG function was assigned to 66.1% of all predicted coding sequences (CDS). We performed a Fisher’s exact test to compare the frequency of each COG category in baseline and SDD isolates. This analysis showed that the distribution of genes to COG categories was similar between isolates from both study periods (Figure S1C, Table S3).

**Figure 2.**
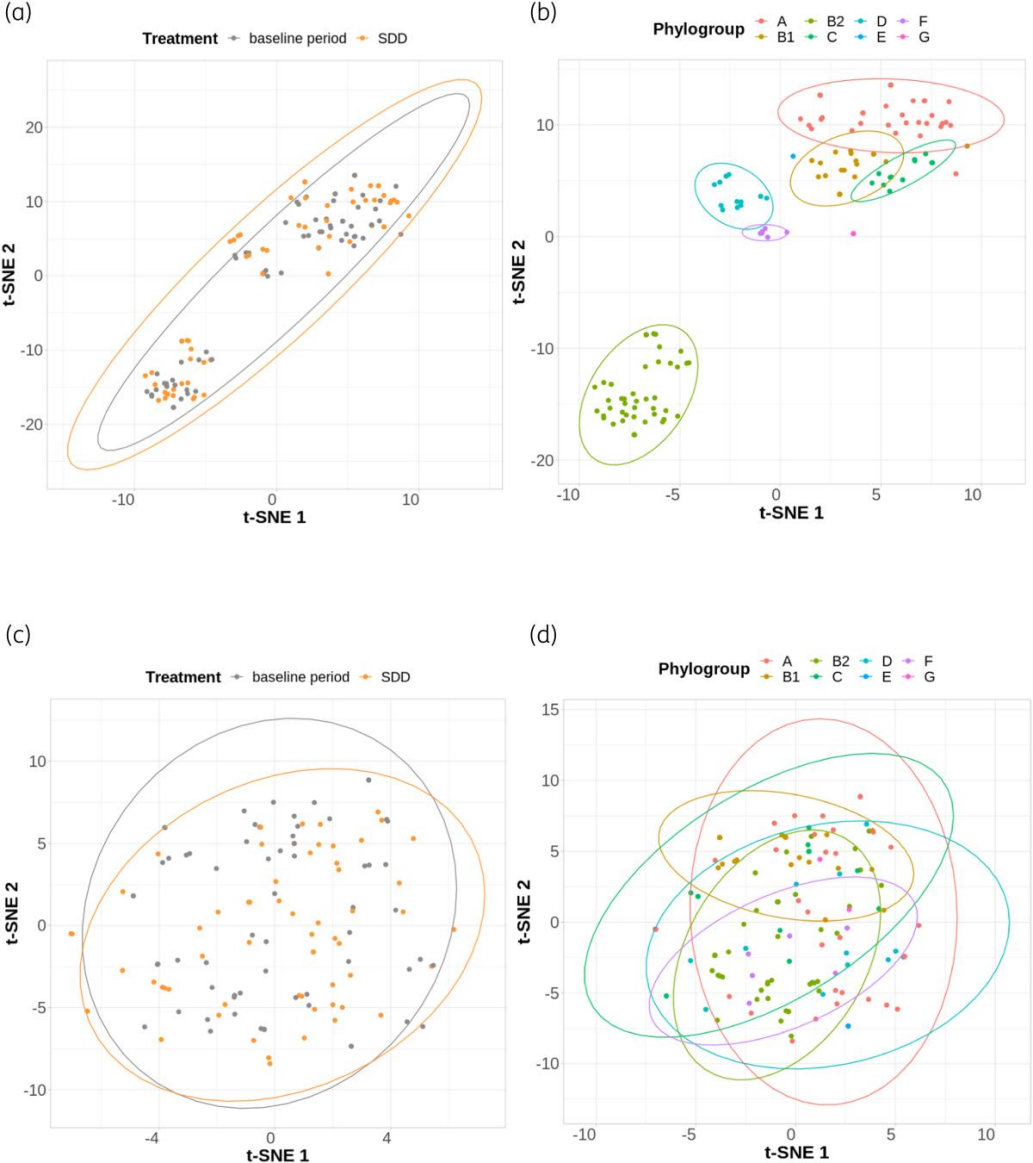
t-SNE plots representing Jaccard distances between complete accessory genomes (a and b) and predicted plasmidomes (c and d) for 129 ESBL-*E. coli* genomes included in this study. Jaccard distances were calculated using gene presence/absence.

Next, we explored the diversity in total accessory gene content of individual isolates and its association with phylogroup, hospital and treatment by using PERMANOVA (Figure 2a and b, Table S1). The accessory genome composition was strongly associated with the isolate’s phylogroup (*R*^2^ = 0.439, *P* value = 0.001), and its interaction with the geographical location (*R*^2^ = 0.10, *P* value = 0.006). There was no significant effect of SDD in the accessory genome composition (*R*^2^ = 0.01, *P* value = 0.149).

The plasmidome is important for niche adaptation in *E. coli*^36^ and other gut bacteria.^37^ Consequently, we also compared predicted plasmidome compositions across study periods. The median of plasmidome sizes for baseline isolates was 196 520.5 bp (IQR = 144 476.5–277 834.5) and of 216 196.0 bp (IQR = 141 770.0–293 638.8) for SDD isolates, which was not statistically different (*P* value = 0.92, Wilcoxon rank-sum test, Figure S2A). Additionally, the median number of unique plasmids per isolate, as predicted by gplas2^38^ reconstructions, was 4 in isolates from both study periods (*P* value = 0.92, Wilcoxon rank-sum test, Figure S2B). Sizes and copy number of individual plasmids also followed expected distributions in both study periods^39^ (Figure S2C).

The use of SDD was also not associated with plasmidome variation (*R*^2^ = 0.011, *P* value = 0.075, PERMANOVA), but the interaction between phylogroup and the ICU explained the largest fraction of plasmidome variation (*R*^2^ = 0.154, *P* value = 0.002, PERMANOVA) (Figure 2c and d, Table S2).

Since the ICU had a significant effect on the plasmidome composition, we wanted to evaluate if this was related to the fact that highly specific plasmid (or clones) were persistent over time in each ICU. For this, we predicted individual plasmids using gplas2, and clustered these plasmid predictions based on MASH distances (Figure S3A and S3B). A total of 558 plasmids were predicted in 128 isolates. Of these, 257 plasmids (46%) were grouped into 65 clusters composed of highly similar plasmids (MASH distance < 0.01), and 37% of these clusters were exclusively composed of plasmids isolated from a single hospital, while 63% included plasmids from multiple hospitals. Moreover, 41.6% (*n* = 10) of plasmid clusters that were recovered in single hospitals were found in multiple clones (PopPUNK clusters) (Figure S3C, Data S2).

### A putative mobile genomic element encoding a tobramycin resistance gene was more prevalent in isolates from SDD-treated patients

A total of 100 unique ARGs were found in the entire dataset (Data S3). Isolates obtained during baseline treatment contained a similar number of acquired ARGs (median = 12, IQR = 8–14) as those of SDD (median = 11, IQR = 7–13) (*P* value = 0.27, Wilcoxon rank-sum test) (Figure S4). When subclassifying ARG by antibiotic class, we found a higher number of aminoglycoside resistance genes in baseline isolates (median = 3, IQR = 1.25–4) than in SDD isolates (median = 2, IQR = 1–3) (*P* value = 0.03, Wilcoxon rank-sum test). For other ARG classes, no significant differences across study periods were found.

We then compared the occurrence of individual ARGs (Figure S5) in both study periods. A total of 12 ARGs had an absolute difference in prevalence larger than 10% across study periods. Out of these, six genes were more frequent in baseline isolates, including two that code for beta-lactamases—*bla*_EC-15_, *bla*_TEM-1_—two genes that provide resistance to streptomycin [*aph(6)-ld, aph(3’)-lb*], and two ARGs that provide resistance to sulfonamide (*sul2*) and tetracyclin (*tet(A)*). Additionally, two genes were more frequent during SDD, namely *aac(6’)-Ib-cr5* and *qnrS1.*

**Figure 3.**
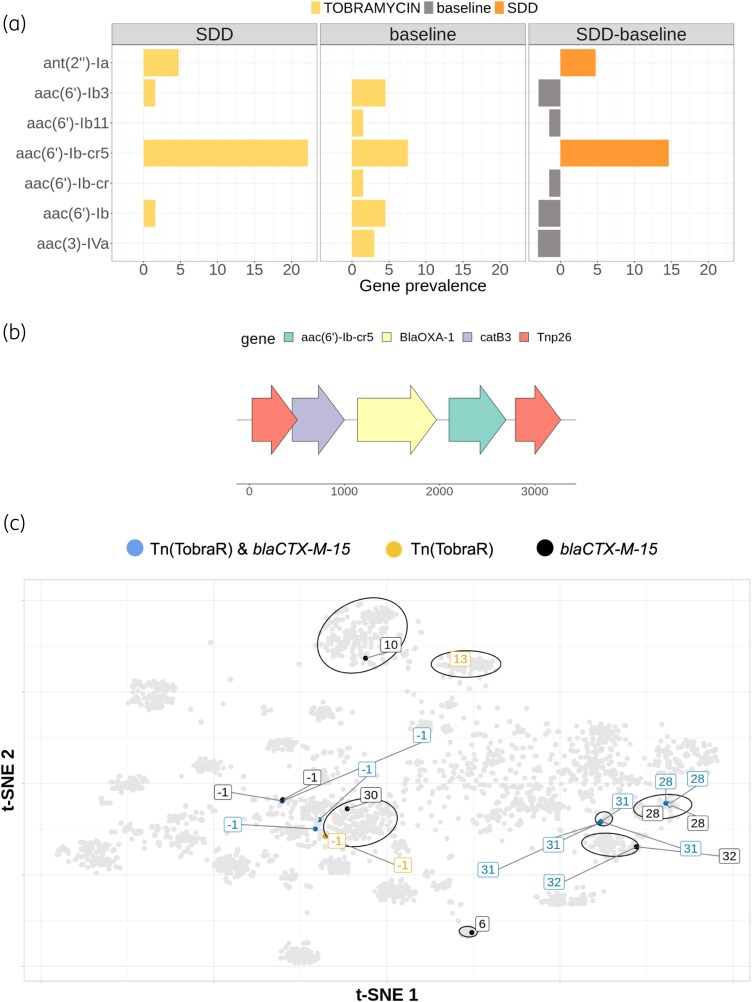
(a) Prevalence of seven ARGs predicted to provide resistance against tobramycin, one of the components of SDD treatment, in isolates from baseline (left) and SDD (centre) patients. The difference in prevalence between the two study periods is displayed on the most right panel. (b) Putative transposon carrying the *aac(6’)-Ib-cr5* gene, termed Tn(TobraR). (c) t-SNE plot in which each dot represents an *E. coli* plasmid. Grey dots represent complete plasmids obtained from NCBI database (*n*∼4500). Coloured dots represent plasmid predictions of isolates from patients that received SDD treatment and that carry Tn(TobraR), *bla*_CTX-M-15_ or both. Labels and ellipses depict different plasmid types, obtained with mge-cluster (v1.1). Labels equal to [−1] correspond to plasmid not assigned to any plasmid type.

The *aac(6’)-Ib-cr5* encodes an aminoglycoside 6'-N-acetyltransferase, which is predicted to confer resistance to tobramycin, one of the components of SDD.^1^ This gene was detected in 5/66 (7.5%) isolates during baseline and in 14/63 (22.2%) isolates during SDD. For other tobramycin-resistance genes, the absolute differences in prevalence between the baseline and SDD periods were below 5% (Figure 3a). To evaluate potential target-site resistance, we screened the short reads for the 16S rRNA *rrsB* T1406A mutation previously linked to tobramycin resistance^40^ and found no evidence of this variant at appreciable frequency in any isolate (ALT_AF ≤0.19%; Data S4).

In the majority of SDD isolates (*n* = 13/14, 92.9%), *aac(6’)-Ib-cr5* was encoded in conjunction with *bla*_OXA-1_ and with *catB3* on a ∼2.2 Kb contig (Figure 3b), which was flanked by IS26 elements (Figure S6). This suggests that this element is a potentially mobile composite transposon, termed from here on Tn(TobraR). Tn(TobraR) was detected in 13 different patients across four hospitals, namely AN (*n* = 1), CD (*n* = 3), LB (*n* = 7) and UZ (*n* = 2), indicating that its occurrence was not restricted to a single study site. The median time between the start of SDD and isolation of Tn(TobraR)-positive isolates was 5 days (IQR = 4–7).

To evaluate if Tn(TobraR) was found in different plasmid backbones, we used mge-cluster^41^ to assign the plasmid predictions generated by gplas2 to existing *E. coli* plasmid clusters (see Materials and methods). We found Tn(TobraR) in four distinct plasmid backbones (Figure 3c), namely cluster 13 (*n* = 1), 28 (*n* = 2), 31 (*n* = 4) and 32 (*n* = 1), and also in five different plasmids that were not assigned to a previously existing plasmid type, supporting the hypothesis that Tn(TobraR) can actually move independently. Additionally, this element was found in isolates with different chromosomal backgrounds, including phylogroups B2 (*n* = 9, most ST131), C (*n* = 1), F (*n* = 1) and A (*n* = 2) (Figure S7A). Moreover, when querying a database composed of more than 1300 publicly available *E. coli* genomes, we found the Tn(TobraR) in 63 additional genomes from six different phylogroups (Figure S7B). Notably, in SDD isolates, we observed that the ARGs that compose the Tn(TobraR) co-occurred with *bla*_CTX-M-15_ in the same plasmid significantly more frequently than expected by chance (Figure S8A, Table S4). However, this was not the case in baseline isolates (Figure S8B, Table S5).

Finally, we evaluated the prevalence of genes coding for carbapenem resistance. A total of four isolates were predicted to have a carbapenemase gene, all of which were obtained from SDD-treated patients. One of these isolates carried a *bla*_OXA-48_ gene in an ST295 background; two isolates from the same patient, belonging to ST410, coded a *bla*_OXA-181_ gene; and *bla*_VIM-1_ was found in an ST1193 isolate.

## Discussion

In this study, we compared 129 ESBL-*E. coli* genomes, 63 from ICU patients receiving SDD prophylaxis and 66 from those not receiving SDD. Overall, there was no evidence observed for an SDD-associated shift in the population structure, pangenome or resistome composition of colonizing ESBL-*E. coli* during the short exposure window captured here. Nevertheless, a specific aminoglycoside resistance element was found more frequently among isolates from SDD-treated patients, warranting careful interpretation.

A strength is the multi-centre design of the study, with isolates from five ICUs in three countries. Because antibiotic practices and endemic *E. coli* lineages and plasmids can differ markedly between ICUs, sampling across five centres reduces the risk that centre-specific clones or plasmids drive apparent treatment-associated signals and supports broader inference across settings. Moreover, in contrast to many similar studies,^42–46^ 96% of the isolates represented intestinal carriage isolates, rather than being recovered from clinical samples. This design avoids clinical bias and offers valuable insight into the genetic and resistome features of *E. coli* isolates colonizing the gut.

The limited impact of SDD on overall pangenome composition was unexpected when considering that SDD alters the microbial composition of the gut,^17,18^ potentially changing the interaction networks that occur in the microbiota leading to new metabolic challenges for *E. coli.*^47^

The absence of important differences in the pangenome can have several potential explanations. First, it is possible that the adaptation of ESBL-*E. coli* to the new gut ecology induced by SDD is not mediated by the acquisition/loss of certain genes, but rather by changes in gene expression patterns. These changes cannot be detected by our analysis, which solely relies on gene content comparisons. Supporting this hypothesis, a study showed global transcriptional rewiring when switching *E. coli* from auxotrophic to prototrophic growth.^48^ Another study demonstrated that auxotrophies can be rescued by short peptides encoded in small ORFs,^49^ which annotation tools might miss. Second, it is also possible that the duration of SDD was not sufficient to cause an impact in the community structure of *E. coli*. Third, isolates from SDD-treated patients were collected after a median of 4 days of exposure, whereas baseline isolates were collected after a median of 6 days following study inclusion. Because microbial colonization dynamics in hospital environments can change rapidly due to continuous exchange between patients and their surroundings, differences in sampling time could influence the detection of transient strains or mobile genetic elements and complicate attribution of modest ARG differences to SDD alone.^50^ Finally, the cross-sectional design of the study, together with the availability of only a single ESBL-positive isolate per patient in most cases, may have limited our ability to capture within-patient temporal dynamics, including transient or early-colonizing strains and changes in the relative abundance of distinct *E. coli* subpopulations. Future research should ideally collect multiple isolates (or faecal samples) over longer periods of time from the same patients, including also non-ESBL-*E. coli*.

The interplay between phylogeny and the ICU explained the largest fraction of variance observed in the accessory genome and plasmidome of isolates. The strong association between phylogeny and the accessory genome of *E. coli* has already been described in.^36,51^ The effect of the ICU could be explained by postulating that each ward constitutes its own ecosystem, in which particular plasmids and clones persist over time, with the ability to spread to different patients. In line with this hypothesis, recent studies suggest that plasmids carrying carbapenem resistance genes present ‘geographical signatures’ that relate them to particular healthcare settings.^52^ Moreover, the long-term persistence of clones and plasmids in clinical environments is more common than originally thought.^50,53–56^ A patient admitted to an ICU can be colonized by bacteria contaminating this environment in less than 6 days.^50^ On the broader scale, particular plasmids have also been associated with specific countries.^57,58^

We observed minor differences between the resistomes of SDD and baseline isolates. While six different resistance genes were more prevalent in baseline isolates, a potentially mobile genetic element composed of a tobramycin resistance gene and two additional ARGs, flanked by IS26 elements, was more frequently found in SDD isolates. An identical genetic element was reported in other studies,^59,60^ but its mobility as an independent unit was never tested. It is well known that IS26 plays a crucial role in the dissemination of resistance genes among Enterobacteriaceae in the clinical environment.^61–63^ IS26 catalyses a highly efficient conservative transposition reaction that allows the incorporation of ARGs preferably into replicons that contain a pre-existing copy of this element.^64,65^ This mechanism could lead to the formation of arrays of in-tandem resistance genes, also referred to as resistance islands.^61^ In line with this, we observed that the tobramycin-resistant transposon identified in this work frequently co-occurred with a *bla*_CTX-M-15_ gene in multiple plasmid backbones that reside in distinct *E. coli* clones. This raises the possibility that, if SDD were to select for this transposon, it could facilitate the formation of resistance islands that accommodate multiple ARGs. Although we focused on IS26-associated mobilization, prophages and phage–plasmid hybrids can also contribute to AMR gene dissemination,^66–69^ but we did not systematically annotate prophage regions in this dataset.

However, the interpretation of these observations should be cautious. First, we had insufficient isolates to perform multivariable analyses correcting for population structure, such as bacterial genome-wide association studies (GWAS).^70^ Second, data on individual therapeutic antibiotic use were not recorded as part of the study; therefore, we could not investigate potential selective pressure exerted by antibiotics other than SDD. Finally, the plasmid background of the tobramycin resistance element was inferred from short-read assemblies and computational plasmid prediction approaches. Although short reads cannot fully resolve plasmid structures, plasmid bins were reconstructed using gplas2, which accurately recovers ARG-carrying plasmids from short-read data, and clustered using mge-cluster, which groups plasmids by shared unitig content consistent with shared backbones. Nevertheless, long-read sequencing would be required to definitively confirm plasmid structures, and the presence of this element across multiple plasmid backgrounds should therefore be considered suggestive.

Point prevalence surveys within the R-GNOSIS ICU study, including 10 997 respiratory and rectal samples found that phenotypic resistance to colistin was rare,^13^ despite this being one of the antibiotics administered in SDD. In concordance with this result, no *mcr* genes were predicted based on WGS data, and only four isolates with phenotypic resistance were reported. Nevertheless, phenotypic resistance was determined using the *E*-test method, which has limited predictive accuracy according to Galani et al.^71^

From an antimicrobial stewardship perspective, the overall similarity in colonizing ESBL-*E. coli* pangenome/resistome composition between study periods is reassuring. The putative transposon detected in 13 SDD-subjected *E. coli* isolates from 13 different patients across four hospitals should be contextualized within the setting of the main study, in which 2082 patients were treated with SDD. No evidence for increased antibiotic resistance was found during the SDD period based on twice weekly surveillance culturing as compared with baseline patients.^13^ Surveillance culturing should remain part of the SDD regimen, to monitor the occurrence of colistin- and or tobramycin-resistant bacteria over time. When increases in resistance are detected/suspected, genotypic testing may be used to investigate potential increases in relevant bacterial clones, plasmids or resistance elements.

Overall, our study constitutes the first WGS-based analysis of the potential effects of SDD on the pangenome composition of a PPMO. Despite the limitations in sample collection, our results suggest that SDD treatment has limited effects in the accessory genome, plasmidome and resistome compositions of ESBL-*E. coli*.

## Supplementary Material

dkag223_Supplementary_Data

## Supplementary material

Supplementary figures, tables and data are available at *JAC* Online.
