# Supplementary material for: Impact of selective digestive decontamination on the pangenome composition of ESBL-E. coli
Source: J Antimicrob Chemother. 2026 Jun 23;81(7):dkag223. doi: 10.1093/jac/dkag223 (PMC13287527; doi:10.1093/jac/dkag223)
Supplement: dkag223_Supplementary_Data [file dkag223_supplementary_data.pdf]

Supplementary Materials

Supplementary Data

Supplementary data can be downloaded from: https://doi.org/10.5281/zenodo.18962610

Supplementary Methods

Plasmidome analysis and plasmid reconstructions

The plasmidome of each genome was defined as all plasmid-predicted contigs identified by using plasmidEC[1] (v1.3). Similar to previously described, plasmidomes were annotated with Bakta, and Jaccard distances between these were calculated based on the presence/absence gene matrix generated by Panaroo[2].

Individual plasmids were reconstructed using gplas2[1] (v1.0). Distances between all plasmid predictions were obtained using MASH[3] (v2.2.2) with k-mer length of 21, and a sketch size of 10,000. Clusters of highly similar plasmids were obtained by creating a network in which connections between plasmids were drawn if their MASH distance was below 0.01.

Clusters of plasmids backbones were created using mge-cluster[4] (v1.1) and clusters numbers were assigned based on the existing *E. coli* database, which can be accessed at: https://doi.org/10.6084/m9.figshare.21674078.v1.

Estimation of plasmid copy number

After short-read assembly with unicycler, each contig is assigned a relative coverage value. We used all unitigs that unambiguously aligned to a single replicon to calculate the mean relative coverage of each plasmid. Duplicated contigs, aligning to more than one location of the genome, were left out of these calculations.

Genomic context reconstruction and read-based detection of the *rrsB* T1406A mutation

To characterize the genomic context of the tobramycin resistance transposon and of $bla_{\text{CTX-M-15}}$, we manually inspected the assembly graph (Unicycler's GFA; nodes representing contigs and edges representing their connectivity) and extracted the nodes adjacent to the target loci. These neighbouring nodes were annotated by BLASTN[5] against the ISFinder[6] database (identity ≥99%,

query coverage ≥95%). We used the assembly graph rather than the linear assembly because insertion sequences are repetitive and may be collapsed in short-read assemblies into short, high-coverage contigs with limited flanking context; the graph representation preserves connectivity needed to evaluate the local neighbourhood around IS-associated nodes.

To evaluate the presence of the 16S rRNA mutation T1406A in *rrsB* (previously associated with tobramycin resistance), raw reads were aligned against the *E. coli rrsB* reference sequence obtained from the CARD database (model ID 39992). Reads were mapped using BWA-MEM[7] (v0.7.19)and alignments were sorted and indexed using SAMtools[8] (v1.23). Base counts and allele frequencies at position 1406 were extracted from the read pileup after applying minimum mapping quality (MQ ≥20) and base quality (BQ ≥30) thresholds, and the alternative allele frequency (ALT_AF) was calculated as ALT/(REF+ALT).

References

1. Paganini JA, Kerkvliet JJ, Vader L, et al. PlasmidEC and gplas2: an optimized short-read approach to predict and reconstruct antibiotic resistance plasmids in Escherichia coli. Microb Genomics. 2024;10(2):001193.

2. Tonkin-Hill G, MacAlasdair N, Ruis C, et al. Producing polished prokaryotic pangenomes with the Panaroo pipeline. Genome Biol. 2020 Jul 22;21(1):180.

3. Ondov BD, Treangen TJ, Melsted P, et al. Mash: fast genome and metagenome distance estimation using MinHash. Genome Biol. 2016 Jun 20;17(1):132.

4. Arredondo-Alonso S, Gladstone RA, Pöntinen AK, et al. Mge-cluster: a reference-free approach for typing bacterial plasmids. NAR Genomics Bioinforma. 2023 Sep 1;5(3):lqad066.

5. Altschul SF, Gish W, Miller W, et al. Basic local alignment search tool. J Mol Biol. 1990 Oct 5;215(3):403–10.

6. Siguier P, Perochon J, Lestrade L, et al. ISfinder: the reference centre for bacterial insertion sequences. Nucleic Acids Res. 2006 Jan 1;34(suppl_1):D32–6.

7. Li H, Durbin R. Fast and accurate short read alignment with Burrows-Wheeler transform. Bioinformatics. 2009 Jul 15;25(14):1754–60.

8. Danecek P, Bonfield JK, Liddle J, et al. Twelve years of SAMtools and BCFtools. GigaScience. 2021 Feb 16;10(2):giab008.

Supplementary Tables

Supplementary Table S1. Results of PERMANOVA analysis to model the variance observed in accessory genome composition. The '*' symbol indicates interaction term between variables. Treatment codes for SDD vs baseline.

Model nr.	Explanatory variables	Df	F.Model	R2	Pr(>F)
1	Phylogroup	7	13.5281039	0.4390271864	0.001
2	Hospital	4	1.216606935	0.03776334787	0.154
3	Treatment	1	1.365392476	0.01063676471	0.149
4	Phylogroup	7	13.7654975	0.4390271864	0.001
	Treatment	1	1.484859951	0.00676529839	0.102
	Phylogroup*Treatment	5	1.327693956	0.03024610429	0.054
5	Phylogroup	7	14.31769118	0.4390271864	0.001
	Hospital	4	1.430689111	0.02506834405	0.054
	Phylogroup*Hospital	18	1.296643498	0.102238266	0.006
6	Treatment	1	1.372662651	0.01063676471	0.143
	Hospital	4	1.2108664	0.03753202137	0.148
	Treatment*Hospital	4	0.9581897992	0.0297000561	0.532

Supplementary Table S2. Results of PERMANOVA analysis to model the variance observed in plasmidome composition. The '*' symbol indicates interaction term between variables. Treatment codes for SDD vs baseline.

Model nr.	Explanatory variables	Df	F.Model	R2	Pr(>F)
1	Phylogroup	7	2.262016978	0.115717622	0.001
2	Hospital	4	1.182226021	0.03673537126	0.099
3	Treatment	1	1.413281789	0.01100572908	0.072
4	Phylogroup	7	2.311171098	0.115717622	0.001
	Treatment	1	1.346775064	0.009633063636	0.083
	Phylogroup*Treatment	5	1.456516246	0.05209004107	0.003
5	Phylogroup	7	2.362907434	0.115717622	0.001
	Hospital	4	1.346915624	0.03769251651	0.016
	Phylogroup*Hospital	18	1.222732122	0.1539779659	0.002
6	Treatment	4	1.18561335	0.03673537126	0.085
	Hospital	1	1.528465622	0.01183960017	0.042
	Treatment*Hospital	4	0.9567051965	0.02964281785	0.592

Supplementary Table S3. Fisher's test results for abundance of different COG types across study periods.

COG	p.value	Confidence interval	OR	Occurence SDD	Occurence baseline period	Not occurence SDD	Not occurence baseline period
J	0.9852496345	c(0.975789492902574, 1.02425758983737)	0.9997054997	13722	14429	200445	210716
K	0.6056192133	c(0.972489422695379, 1.0163625709264)	0.9941842403	16759	17713	197408	207432
F	0.9719039845	c(0.965246095111443, 1.03455419087637)	0.9993147717	6486	6823	207681	218322
Q	0.9366409714	c(0.951155658340286, 1.05601402039197)	1.002227294	2819	2957	211348	222188
G	0.6018505376	c(0.985794356531915, 1.02508083454845)	1.005237299	22074	23097	192093	202048
I	0.6933838823	c(0.974037533976659, 1.0405276915715)	1.006727248	7196	7516	206971	217629
E	0.8130938607	c(0.982364983479942, 1.02289454010526)	1.00244109	20432	21432	193735	203713
C	0.9634520666	c(0.978245285628295, 1.02330688097101)	1.000524396	16079	16895	198088	208250
M	0.8085665345	c(0.974179799777156, 1.02052782175703)	0.9970788874	15003	15815	199164	209330
O	0.906893496	c(0.969951310487423, 1.02736934293829)	0.9982685969	9547	10053	204620	215092
R	0.8888010572	c(0.972289137890958, 1.02457843874712)	0.9980901027	11625	12243	202542	212902
U	0.8477019785	c(0.952154529987101, 1.04089787909678)	0.99555203	3884	4101	210283	221044
T	0.9945601485	c(0.973350466549645, 1.02703215378781)	0.9998562239	11065	11634	203102	213511
P	0.9345003041	c(0.976456561365551, 1.026244226516)	1.001042395	13000	13653	201167	211492
H	0.8402077964	c(0.976601630259693, 1.02963648049424)	1.002776834	11422	11976	202745	213169
S	0.9486169264	c(0.96991901626744, 1.03340134536923)	1.001155743	7806	8197	206361	216948
D	0.8987001219	c(0.949242121483854, 1.06140674472677)	1.003776748	2471	2588	211696	222557
V	0.9926284441	c(0.963969778713874, 1.03672824250224)	0.9996686522	5875	6178	208292	218967
L	0.5763402559	c(0.960966481318246, 1.02237950636188)	0.9912170141	8154	8645	206013	216500
X	0.0649580104	c(0.996571842983943, 1.11875968712521)	1.055920887	2362	2353	211805	222792
N	0.1746329295	c(0.934173122750306, 1.01245725611287)	0.9725566337	4723	5102	209444	220043
W	0.970997954	c(0.928852378768879, 1.07256736623477)	0.9981455043	1485	1564	212682	223581
A	0.6192491546	c(0.740511742054748, 1.68314621513269)	1.115649109	52	49	214115	225096
Z	1	c(0.779773374511084, 1.29089404928233)	1.003476705	126	132	214041	225013

Supplementary Table S4. Co-occurrence of ARGs in the same plasmid in SDD isolates. Co-occurrences with a p-value smaller than 0.01 were considered significant.

ARG 1	ARG 2	ARG 1 Occurence	ARG 2 Occurence	Co-occurence	Prob. Co-occurence	Expected co-occurence	p-value
aac(3)-IId	blaTEM-1	7	21	4	0.014	1.4	0.03039
aac(3)-IId	mph(A)	7	24	4	0.016	1.6	0.04957
aac(3)-IIe	aac(6')-Ib-cr5	11	13	4	0.013	1.4	0.0312
aac(3)-IIe	blaCTX-M-15	11	18	8	0.019	1.9	2.00E-05
aac(3)-IIe	blaOXA-1	11	14	4	0.015	1.5	0.04108
aac(3)-IIe	catB3	11	14	4	0.015	1.5	0.04108
aac(6')-Ib-cr5	blaCTX-M-15	13	18	10	0.022	2.3	0
aac(6')-Ib-cr5	blaOXA-1	13	14	13	0.017	1.8	0
aac(6')-Ib-cr5	catB3	13	14	13	0.017	1.8	0
aac(6')-Ib-cr5	mph(A)	13	24	7	0.029	3	0.01073
aadA1	aadA2	17	8	5	0.013	1.3	0.00286
aadA1	aadA5	17	25	0	0.04	4.1	1
aadA1	blaCTX-M-15	17	18	0	0.029	3	1
aadA1	dfrA17	17	31	0	0.05	5.1	1
aadA1	mph(A)	17	24	0	0.038	4	1
aadA5	blaCTX-M-15	25	18	9	0.042	4.4	0.00842
aadA5	blaOXA-1	25	14	7	0.033	3.4	0.02319
aadA5	blaTEM-1	25	21	9	0.049	5.1	0.02964
aadA5	catB3	25	14	7	0.033	3.4	0.02319
aadA5	dfrA17	25	31	20	0.073	7.5	0
aadA5	mph(A)	25	24	17	0.057	5.8	0
aadA5	sul1	25	33	21	0.078	8	0
aph(3'')-Ib	aph(6)-Id	19	19	19	0.034	3.5	0
aph(3'')-Ib	blaTEM-1	19	21	10	0.038	3.9	0.00048
aph(3'')-Ib	sul2	19	23	17	0.041	4.2	0
aph(3'')-Ib	tet(A)	19	20	10	0.036	3.7	0.00028
aph(6)-Id	blaTEM-1	19	21	10	0.038	3.9	0.00048
aph(6)-Id	sul2	19	23	17	0.041	4.2	0
aph(6)-Id	tet(A)	19	20	10	0.036	3.7	0.00028
blaCTX-M-15	blaOXA-1	18	14	11	0.024	2.4	0
blaCTX-M-15	catB3	18	14	11	0.024	2.4	0
blaCTX-M-15	dfrA17	18	31	9	0.053	5.4	0.04365
blaCTX-M-15	sul1	18	33	10	0.056	5.8	0.02113
blaOXA-1	catB3	14	14	14	0.018	1.9	0
blaOXA-1	mph(A)	14	24	7	0.032	3.3	0.01802
blaOXA-1	sul1	14	33	8	0.044	4.5	0.03464
blaTEM-1	sul1	21	33	11	0.065	6.7	0.02609
catB3	mph(A)	14	24	7	0.032	3.3	0.01802
catB3	sul1	14	33	8	0.044	4.5	0.03464
dfrA17	mph(A)	31	24	12	0.07	7.2	0.01661
dfrA17	sul1	31	33	16	0.096	9.9	0.00573
mph(A)	sul1	24	33	18	0.075	7.7	0
sul2	tet(A)	23	20	9	0.043	4.5	0.01047

Supplementary Table S5. Co-occurrence of ARGs in the same plasmid in baseline isolates. Co-occurrences with a p-value smaller than 0.01 were considered significant.

ARG 1	ARG 2	ARG 1 Occurence	ARG 2 Occurence	Co-occurence	Prob. co-occurence	Expected co-occurence	p-value
aadA1	aadA2	14	10	4	0.011	1.2	0.02122
aadA1	aadA5	14	22	0	0.025	2.8	1
aadA1	dfrA17	14	29	0	0.032	3.6	1
aadA2	dfrA17	10	29	0	0.023	2.6	1
aadA5	dfrA17	22	29	11	0.051	5.7	0.00601
aadA5	mph(A)	22	20	12	0.035	3.9	1.00E-05
aadA5	sul1	22	31	14	0.054	6.1	8.00E-05
aadA5	sul2	22	38	12	0.067	7.5	0.02302
aph(3'')-Ib	aph(3')-Ia	39	12	9	0.037	4.2	0.00339
aph(3'')-Ib	aph(6)-Id	39	39	39	0.121	13.6	0
aph(3'')-Ib	blaTEM-1	39	41	20	0.127	14.3	0.01618
aph(3'')-Ib	dfrA1	39	4	4	0.012	1.4	0.01324
aph(3'')-Ib	dfrA17	39	29	3	0.09	10.1	0.99989
aph(3'')-Ib	sul2	39	38	32	0.118	13.2	0
aph(3')-Ia	aph(6)-Id	12	39	9	0.037	4.2	0.00339
aph(3')-Ia	blaTEM-1	12	41	9	0.039	4.4	0.00522
aph(3')-Ia	sul2	12	38	9	0.036	4.1	0.0027
aph(6)-Id	blaTEM-1	39	41	20	0.127	14.3	0.01618
aph(6)-Id	dfrA1	39	4	4	0.012	1.4	0.01324
aph(6)-Id	dfrA17	39	29	3	0.09	10.1	0.99989
aph(6)-Id	sul2	39	38	32	0.118	13.2	0
blaCTX-M-27	sul1	4	31	4	0.01	1.1	0.00507
blaTEM-1	dfrA17	41	29	5	0.095	10.6	0.99784
blaTEM-1	sul2	41	38	21	0.124	13.9	0.00333
blaTEM-1	sul3	41	6	5	0.02	2.2	0.02412
blaTEM-1	tet(M)	41	3	3	0.01	1.1	0.04677
dfrA1	sul2	4	38	4	0.012	1.4	0.01188
dfrA17	tet(A)	29	35	4	0.081	9.1	0.9968
mph(A)	sul1	20	31	15	0.049	5.5	0
mph(A)	tet(A)	20	35	10	0.056	6.2	0.04451
tet(A)	tet(B)	35	13	1	0.036	4.1	0.99459

Supplementary Figures

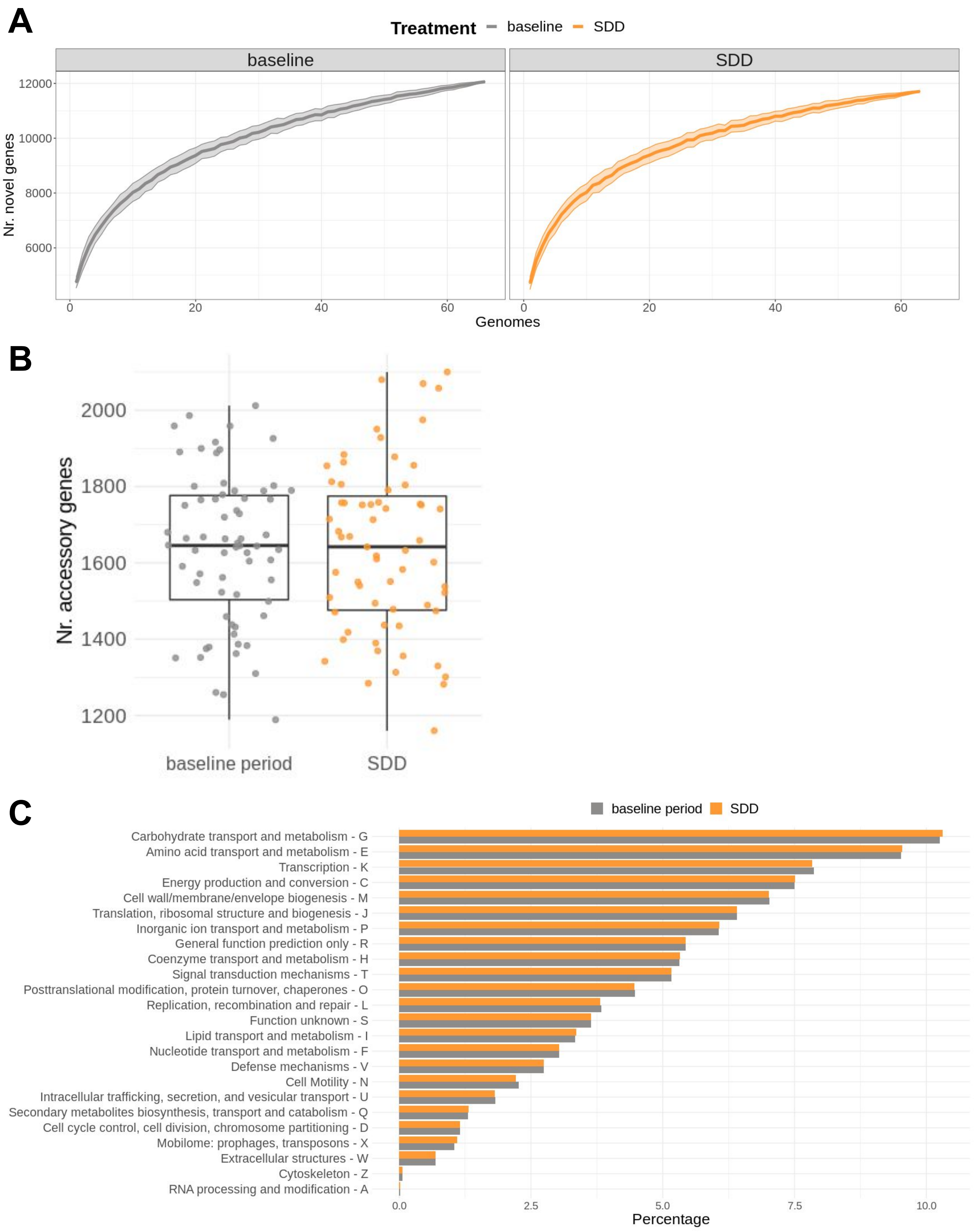

Supplementary Figure S1. A) Pangenome accumulation curves for baseline and SDD isolates. **B)** Number of accessory genes per isolate across different study periods. **C)** Fraction of COG functional categories by study period.

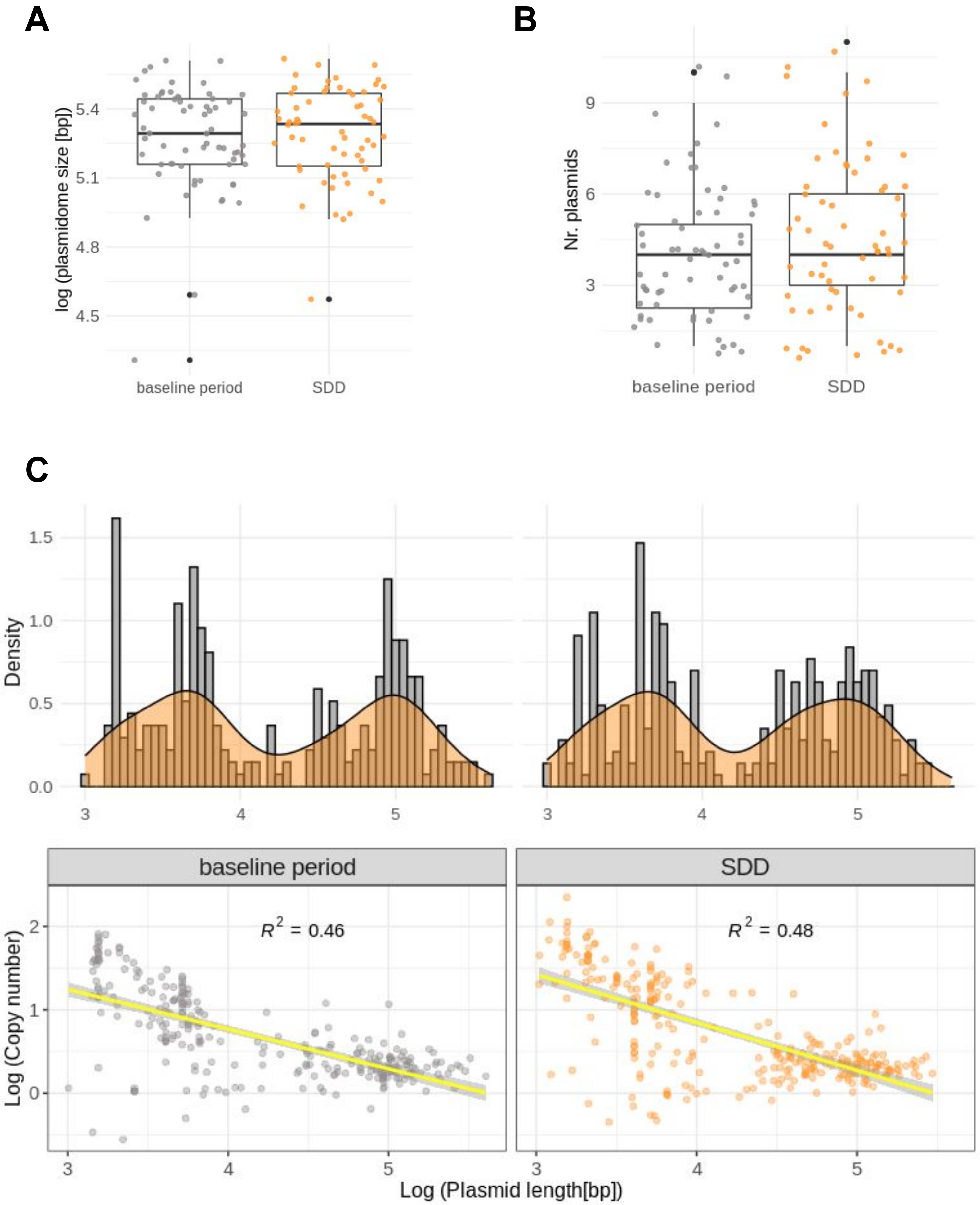

Supplementary Figure S2. A) Total size of predicted plasmidome sorted by study period. This is, the total length of all contigs within an isolate predicted to be plasmid by plasmidEC. **B)** Number of predicted individual plasmids per isolate. Plasmids were predicted using gplas. **C)** Plasmid size vs estimated copy number for individual plasmid predictions

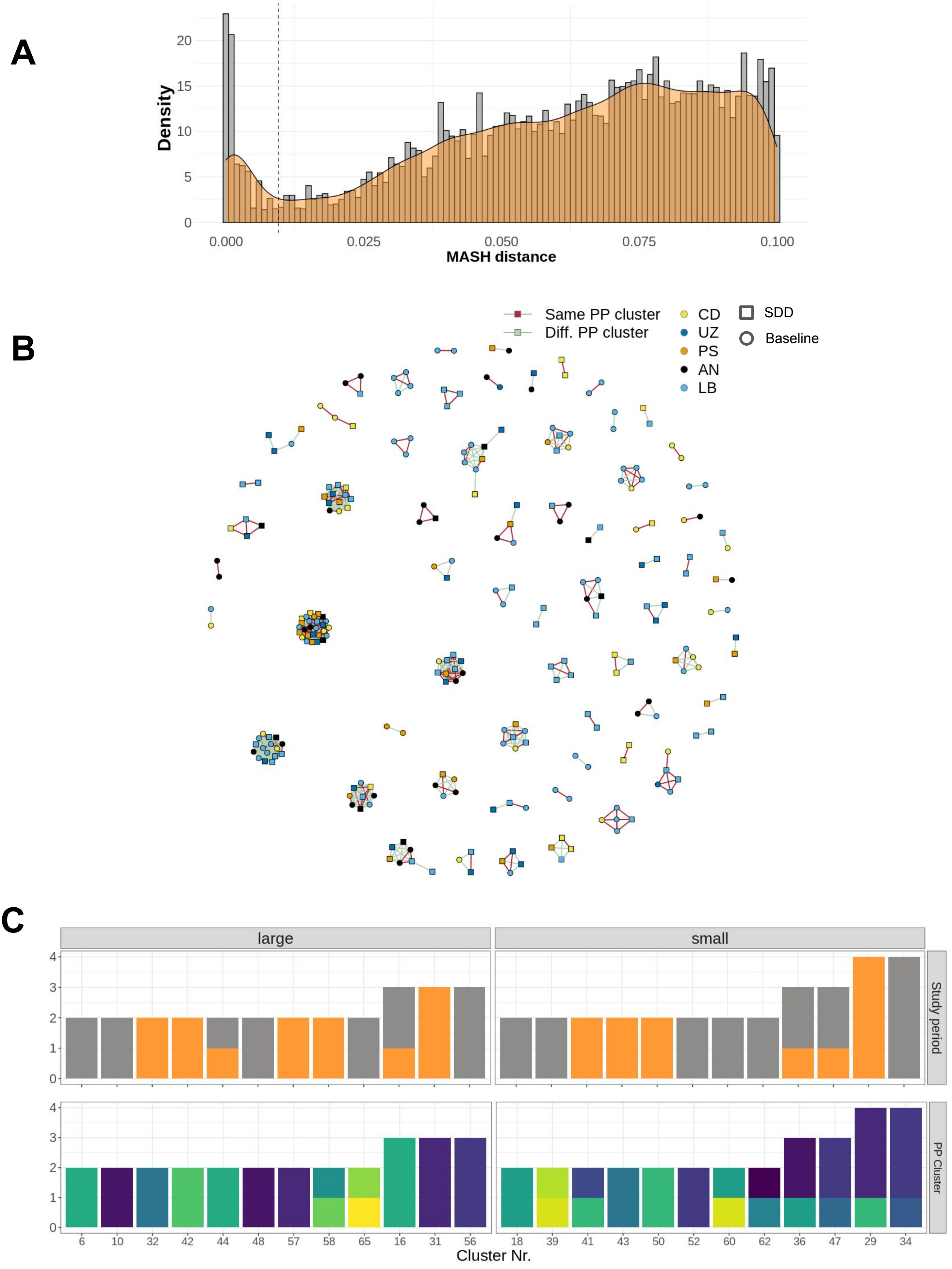

Supplementary Figure S3. A) MASH distances (k=21, s=10,000) for all plasmid-predictions vs plasmid-predictions. Dashed line indicates the cut-off point (distance =0.01) to create network of plasmids. **B)** Network displaying clusters of highly similar plasmids. **C)** For plasmid clusters that were present only in single hospitals, study period in which they were found and the number of distinct PopPUNK clusters is indicated.

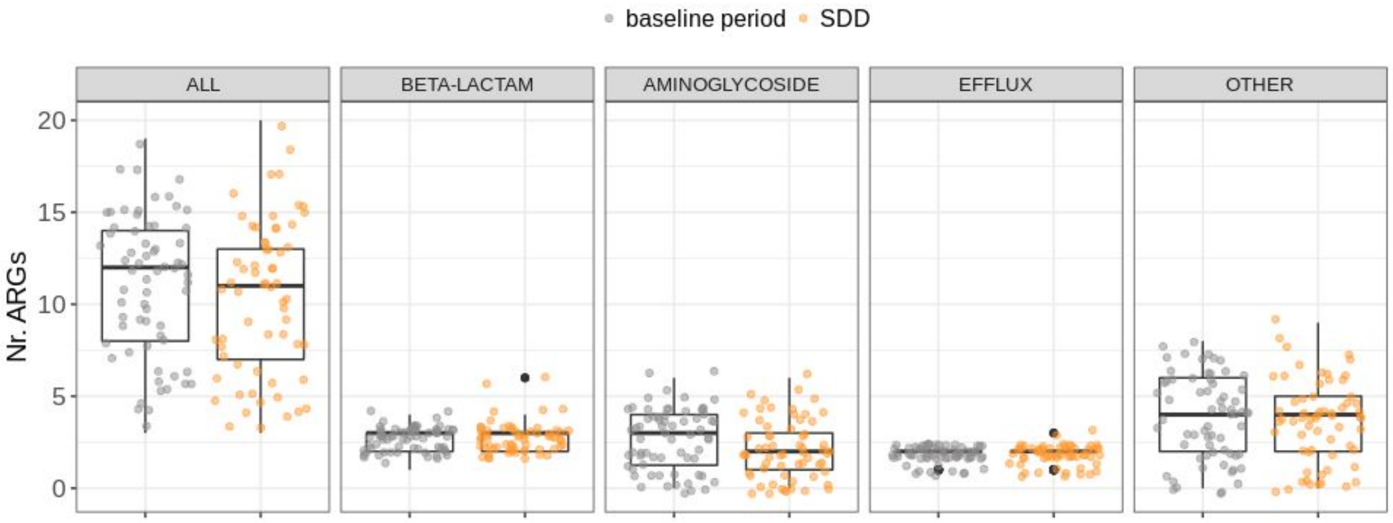

Supplementary Figure S4. Nr. of acquired ARGs per isolate, treatment and ARG type.

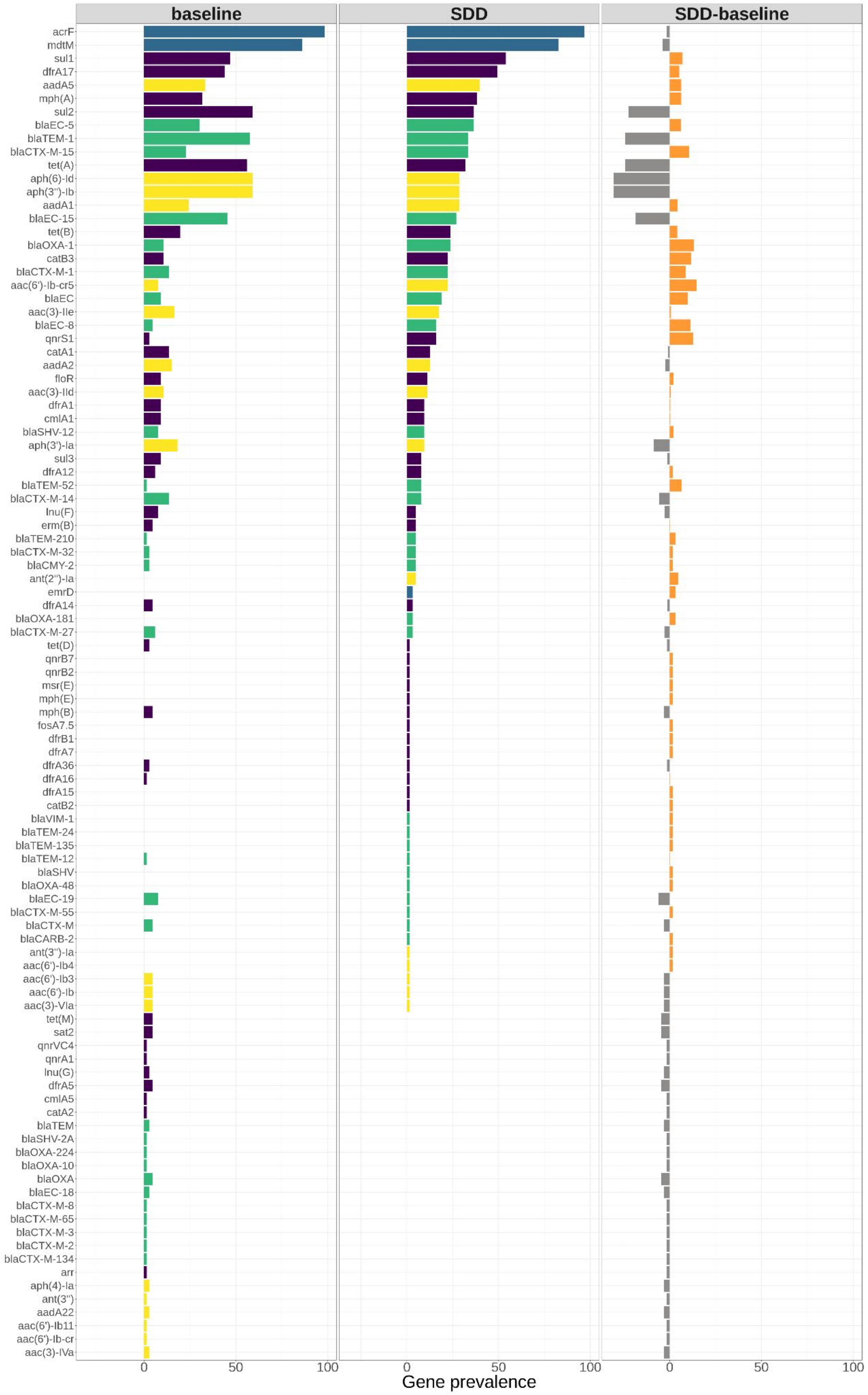

Supplementary Figure S5. The first two panels show the prevalence of all acquired ARGs in SDD and baseline isolates. The third panel shows the absolute difference between these prevalences in SDD and baseline periods.

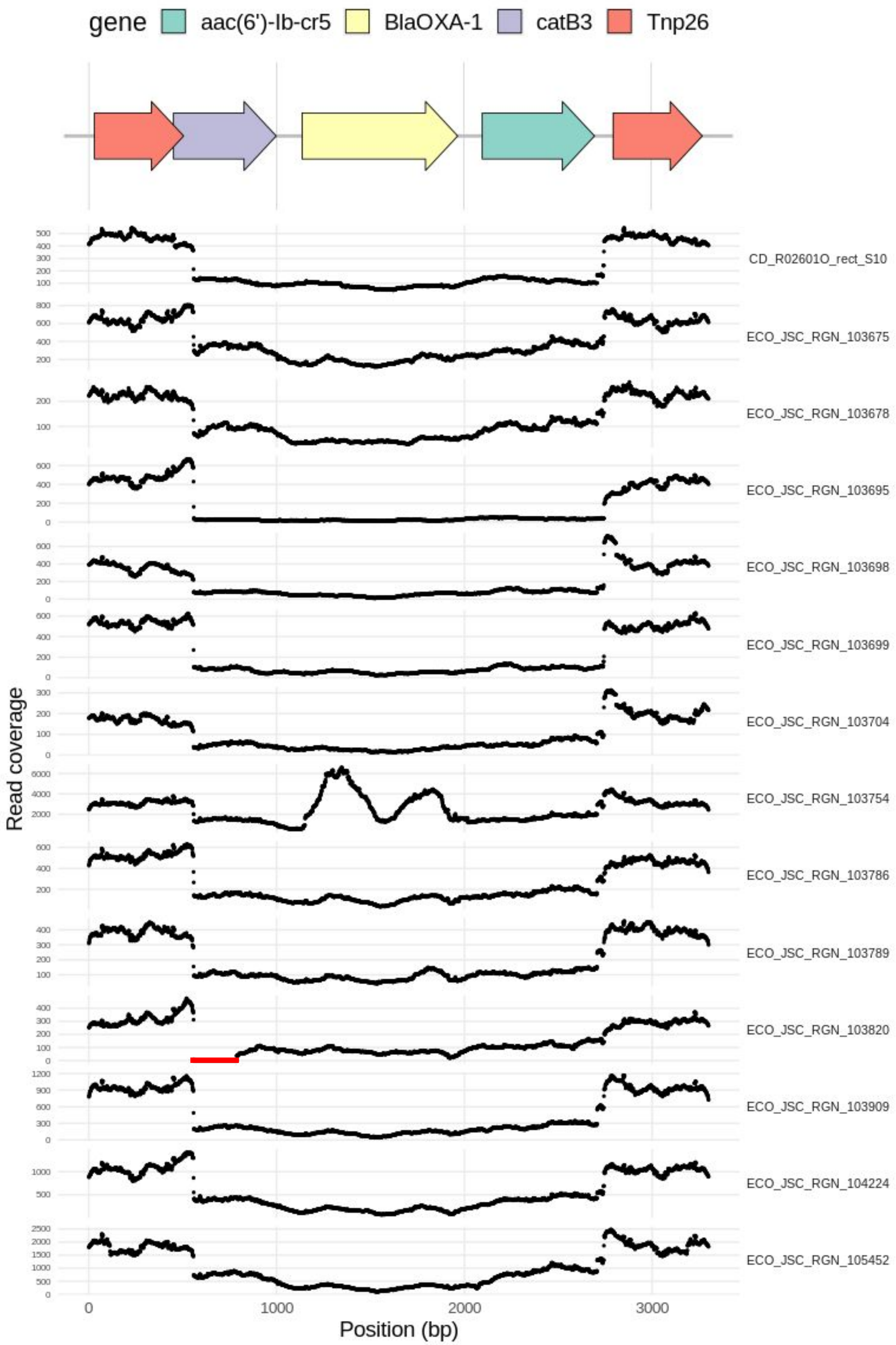

Supplementary Figure S6. Read coverage of each SDD isolate (n=14) that contains the putative transposon carrying the tobramycin resistance gene. Coverage suggests that all SDD isolates, except ECO-JSC-RGN-103820, carry the complete sequence of Tn(TobraR). Red lines indicate regiones with read coverage equal to zero.

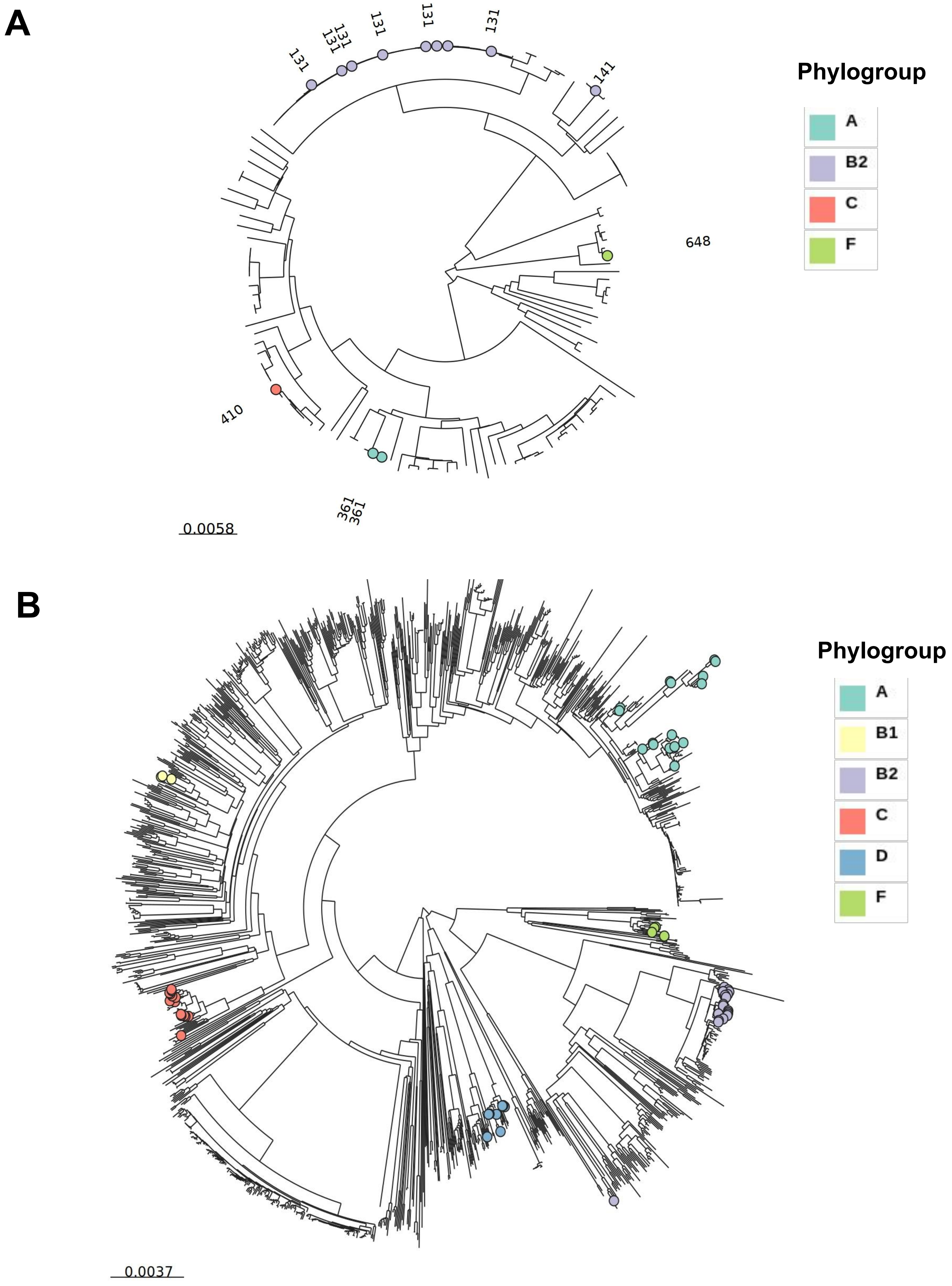

Supplementary Figure S7. A) NJ tree based on core-genome alignment of *E. coli* isolates from the R-GNOSIS study. Leaf labels indicate the sequence type of isolates. **B)** NJ cg-tree based on k-mer presence/absence of 1381 publicly available *E. coli* complete genomes. In both trees, colored nodes indicate the genomes that carry Tn(TobraR) and their corresponding phylogroup.

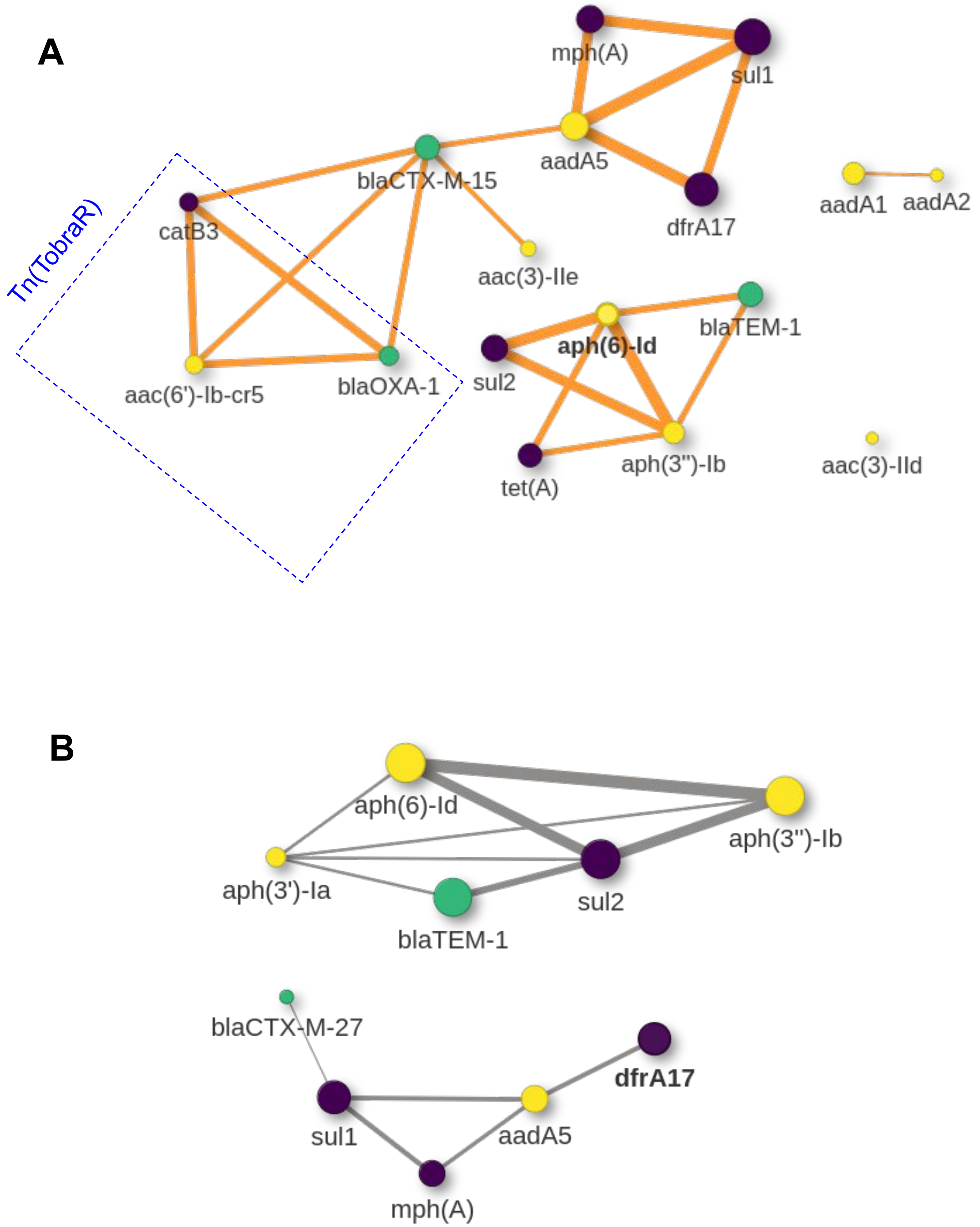

Supplementary Figure S8. A) Co-occurrence network of ARGs in the same plasmid prediction in SDD isolates. **B)** Co-occurrence network of ARGs in the same plasmid prediction in baseline isolates. Only connections with a p-value=<0.01 are drawn.
